# Associations between the prevalence of chronic hepatitis B among people who inject drugs and country‐level characteristics: An ecological analysis

**DOI:** 10.1111/dar.13595

**Published:** 2023-01-04

**Authors:** Anjalee Syangbo, Matthew Hickman, Samantha Colledge‐Frisby, Janni Leung, Jason Grebely, Sarah Larney, Louisa Degenhardt, Adam Trickey

**Affiliations:** ^1^ Population Health Sciences University of Bristol Bristol UK; ^2^ National Drug and Alcohol Research Centre, UNSW Sydney Sydney Australia; ^3^ National Drug Research Institute Melbourne Australia; ^4^ National Centre for Youth Substance Use Research The University of Queensland Brisbane Australia; ^5^ Kirby Institute, UNSW Sydney Sydney Australia; ^6^ Department of Family Medicine and Emergency Medicine University of Montreal Montreal Canada; ^7^ Research Centre of the Hospital Centre of the University of Montreal Montreal Canada

**Keywords:** ecological, hepatitis B virus, people who inject drugs, prevalence

## Abstract

**Introduction:**

Globally, hepatitis B virus (HBV) is a leading cause of liver disease. People who inject drugs (PWID) are at greater risk than the general population of contracting HBV. This risk could depend on societal factors in different countries. We investigated the associations between country‐level chronic HBV prevalence in PWID with national indicators of development and prevalence of HIV and hepatitis C virus (HCV).

**Methods:**

We used global systematic review data on chronic HBV prevalence (hepatitis B surface antigen‐positive) among PWID and country‐level sociodemographic characteristics from online databases. National random‐effects meta‐analysis estimates of HBV prevalence were the outcome in linear regression models testing for associations with country‐level characteristics.

**Results:**

The study included 131,710 PWID from 304 estimates in 55 countries: the pooled HBV prevalence among PWID in the countries analysed was 4.5% (95% CI 3.9–5.1), the highest regional pooled prevalence was in East and Southeast Asia (17.6% [13.3–22.3]), and the lowest was in Western Europe (1.7% [1.4–2.1]). In multivariable models, no indicators of development were associated with HBV prevalence, but there was evidence of positive associations between HBV prevalence in the general population and among PWID, and evidence of HIV and HCV prevalence in PWID being associated with HBV prevalence in PWID: multivariable coefficients 0.03 (95% CI 0.01–0.04); *p* < 0.001, and 0.01 (95% CI 0.00–0.03); *p* = 0.01, respectively.

**Discussion and Conclusions:**

HBV prevalence among PWID was associated with HIV and HCV prevalence among PWID and background HBV prevalence in the general population, highlighting the need for improving harm reduction in PWID and implementation of HBV vaccination, especially where HBV is endemic.

## 1 INTRODUCTION

Hepatitis B virus (HBV), a blood‐borne virus that causes liver disease, primarily hepatocellular carcinoma and cirrhosis, results in substantial morbidity and mortality [1]. Worldwide, chronic HBV is the most common cause of hepatocellular carcinoma, with attributable cases ranging from 50% in regions with low HBV endemicity and up to 70–80% in highly endemic areas [2]. Chronic infection is indicated by the sustained presence of the serological marker, hepatitis B surface antigen (HBsAg), for at least 6 months [3]. The risk of progression from acute to chronic disease is inversely proportional to age; approximately 5% of adults exposed to HBV develop chronic infection. Most of the global population (~88%) live in countries where HBV prevalence is intermediate (2–8%) or high (≥8%), as classified by the World Health Organization [4].

HBV is transmitted by parental or mucosal exposure to HBsAg‐positive bodily fluids, particularly blood. Lifelong protection is provided by a safe and effective vaccine delivered through infant programs. While immunisation coverage has increased considerably in recent years from 30% in 2000 to 85% in 2019, rates vary radically across the globe [3]. Direct, percutaneous inoculation by sharing contaminated injecting equipment, such as needles and syringes, is an important mode of HBV transmission [5]. There are an estimated 15.6 million (95% uncertainty interval 10.2, 23.7 million) people who inject drugs (PWID) across the globe with a higher risk of contracting HBV than the general population of their respective countries [5]. For example, in 2015, 30.3% of newly HBV‐infected individuals in the United States reported injecting drug use as a critical risk factor [4], indicating the importance for further work to address prevention and treatment of HBV infection among PWID. The World Health Organization has set targets for the elimination of hepatitis by 2030 as a public health threat and PWID are a key population to consider if these goals are to be achieved [6].

Recent estimates suggest that 9.1% (95% uncertainty interval: 5.1–13.2%) of PWID suffer from HBV, globally, equating to roughly 1.4 million (0.7–2.4 million) people [5]. About 22.6% of PWID have evidence of past infection [7]. East and Southeast Asia accounts for more than half of all HBsAg‐positive PWID worldwide [5] and 51% of the global IDU‐attributable HBV burden [8], yet the estimated 4.0 million (95% uncertainty interval 3.0, 5.0 million) PWID living there only represent 25% of the global population of PWID [5]. The burden of the HBV epidemic among PWID in different parts of the world may be influenced by country‐level factors.

While previous research has highlighted that country‐level income is associated with HBV prevalence in the general population, there has been limited research into country‐level associations with HBV prevalence among PWID. This ecological analysis used country‐level indicators of a nation's socioeconomic and development status and epidemiological data from our previously published global systematic review to explore whether country‐level measures of disadvantage were associated with chronic HBV prevalence in PWID. Our hypothesis was that countries with higher levels of inequality and poverty would have higher prevalence of HBV among PWID when controlling for general population HBV prevalence, a relationship that would be acting through the unequal distribution of vaccinations and harm‐reduction measures.

## 2 METHODS

### 2.1 Data sources

The data were from a previous, multistage, global systematic review on population sizes and prevalence of HIV, HBV and hepatitis C (HCV) in PWID conducted by Degenhardt et al. [5] (PROSPERO registration numbers CRD42016052858 and CRD42016052853), combined with country‐level sociodemographic characteristics from various sources, described below. The review was completed in 2017 as an update to the last available estimates for viral hepatitis prevalence from 2011 [9]. Full methods have been published elsewhere [5]. The review included journal articles indexed in Medline, Embase and PsycINFO and online reports from government, intergovernmental and non‐governmental organisations published between January 2008 to June 2017, as well as data via expert requests. Some of the main inclusion criteria were: PWID samples greater than 40 individuals, samples that represented the whole PWID population and not just sub‐populations (e.g., prison populations) and, where multiple estimates were available, literature with a higher quality grade and increased geographic coverage took precedence over the recency of the estimate [5]. The inclusion flow diagram of the review by Degenhardt et al. [5] is included as Figure S1, Supporting Information, while a GATHER checklist for this current manuscript is included as Table S1.

### 2.2 Variables

As this analysis focused on chronic HBV, only studies with from the Degenhardt et al. review [5] with data on HBsAg were included. Studies were excluded that looked exclusively at alternative serological markers (such as anti‐HBc, a marker that only indicates past or current infection and not whether the infection is acute or chronic). Most studies reported a percentage of the PWID sample infected with HBsAg. Where this information was omitted, we calculated the percentage using the sample size and the number of HBsAg‐positive individuals.

The values for the country‐level characteristics were obtained from multiple sources. Country‐level HIV and HCV (antibodies) prevalence in PWID were as in the review by Degenhardt et al. [5]. The rest of the indicators: HBV prevalence in the whole population, Gender Inequality Index, Gini coefficient, gross domestic product (billion US$), gross national income (US$), Human Development Index, HepB3 (3‐dose) immunisation coverage among 1‐year‐olds, labour force participation rate (women), prisoners per 100,000 of the whole population, sociodemographic index, urbanisation, women with secondary education and both male and female youth unemployment, were obtained from global online databases. We imputed any missing values using similar sources. For full details on the indicators, source, value, and additional notes, see Tables S2 and S3 (Supporting Information).

### 2.3 Meta‐analysis

Taiwan and the Occupied Palestinian Territory had large amounts of missing country‐level characteristic data and were thus omitted from further analysis. A random‐effects meta‐analysis was conducted using the study sample size and the number of individuals infected with HBsAg to generate a single pooled chronic HBV prevalence estimate per country. This was to allow for studying between‐country variation as opposed to between‐study. A secondary meta‐analysis was undertaken to stratify the sample by UNAIDS region instead of country to generate regional average percentages of those infected by HBsAg.

### 2.4 Country‐level regression

We used general linear models to test for associations between the national estimate of chronic HBV prevalence among PWID (converted to a proportion and logit transformed: [log *p*/(1 − *p*)] [10]) and the country‐level characteristics as the independent variables. Scatter plots of HBV prevalence and country‐level characteristics were generated and superimposed by linear and quadratic curves to visually inspect trends. For each independent variable, both a linear and quadratic univariable model were run and then compared for their fit using the Bayesian Information Criterion and a likelihood ratio test. If the Bayesian Information Criterion values were highly similar, the likelihood ratio test were used to solve any discrepancies. The model with the best fit was subsequently used moving forward. Data were analysed using Stata (version 16.0); source code for this analysis can be shared upon request with the corresponding author.

If HBV prevalence is highly endemic in a particular nation, it is likely that the chronic HBV prevalence in PWID would also be higher as a result. Thus, we included HBV prevalence in the whole population, in addition to the country‐level characteristics, in the multivariable models.

## 3 RESULTS

For this analysis, data on chronic HBV prevalence in PWID was reported in 55 countries, accounting for ~63% of the global population. The study included 131,710 PWID from 304 estimates in total. Greece had the highest number of estimates (*n* = 70, partially due to having separate estimates for different cities/regions) and there were 20 countries with only one estimate. All studies from which estimates were generated were conducted between 2008 and 2017 (Table 1).

**TABLE 1:** Characteristics of estimates included in the analysis, categorised by UNAIDS region

Country of report	Number of estimates	Publication year(s)	Sample size range	% infected HBsAg range*	% infected HBsAg (95% CI)
Australasia (1 country)	2	2009, 2012	382–402	3.0–4.0	3.6 (2.4, 5.0)
Australia	2	2009, 2012	382–402	3.0–4.0	3.6 (2.4, 5.0)
East and Southeast Asia (5 countries)	24	2009–2015	97–1049	2.4–51.6	17.6 (13.3, 22.3)
China	8	2009, 2010, 2011, 2014, 2017	97–1049	2.4–51.6	20.4 (11.4, 31.1)
Korea (Republic of)	1	2013	318	6.6	6.6 (4.1, 9.9)
Myanmar	2	2011	318–1029	12.3–43.1	15.3 (13.4, 17.3)
Thailand	1	2008	1535	30.5	30.5 (28.2, 32.9)
Vietnam	12	2011, 2012, 2015	272–1000	10.7–28.0	14.7 (12.0, 17.5)
Eastern Europe (12 countries)	59	2008–2016	42–9405	0.0–76.8	5.5 (4.0, 7.2)
Azerbaijan	14	2008, 2012	100–300	2.0–14.0	7.9 (5.9, 10.1)
Belarus	6	2015	160–400	0.4–31.9	9.6 (2.5, 20.4)
Bosnia and Herzegovina	2	2012, 2016	120–130	0.0–0.8	0.2 (0.0, 1.5)
Bulgaria	7	2016	661–1258	3.1–9.8	5.7 (3.9, 7.8)
Estonia	3	2010, 2012, 2016	326–351	4.0–76.8	23.1 (0.0, 75.9)
Hungary	6	2012, 2015, 2016	223–666	0.3–2.2	0.9 (0.3, 1.6)
Latvia	6	2016	81–1147	1.6–6.2	1.7 (1.1, 2.5)
Lithuania	1	2016	200	10.5	10.5 (6.6, 15.6)
Moldova (Republic of)	4	2013	115–365	0.0–12.4	4.6 (1.1, 10.3)
Romania	3	2011, 2016	45–522	5.0–10.6	7.3 (3.6, 12.2)
Slovakia	6	2016	42–67	1.7–5.1	2.6 (1.0, 4.8)
Ukraine	1	2016	9405	5.6	5.6 (5.1, 6.0)
Middle East and North Africa (7 countries)	19	2009–2016	40–4694	0.0–8.6	2.9 (2.0, 3.9)
Cyprus	6	2010, 2012, 2016	40–349	0.0–6.1	0.5 (0.0, 1.7)
Israel	1	2010	199	6.3	6.0 (3.2, 10.3)
Lebanon	1	2010	81	2.5	2.5 (0.3, 8.6)
Saudi Arabia	1	2015	378	7.7	7.7 (5.2, 10.8)
Syrian Arab Republic	1	2014	394	0.5	0.5 (0.1, 1.8)
Tunisia	3	2009	62–712	0.0–3.5	2.7 (1.5, 4.1)
Turkey	6	2016	964–4694	3.6–8.6	4.7 (3.5, 6.2)
North America (1 country)	1	2015	462	4.8	4.5 (2.8, 6.9)
United States of America	1	2015	462	4.8	4.5 (2.8, 6.9)
South Asia (7 countries)	66	2008–2016	58–2292	0.0–43.0	6.7 (5.2, 8.2)
Afghanistan	12	2010, 2011, 2012, 2014	96–483	3.2–10.4	6.2 (5.1, 7.4)
Bangladesh	2	2008, 2015	400–561	7.0–2.9	8.4 (6.7, 10.2)
India	27	2008, 2009, 2010, 2011, 2012, 2013, 2014, 2015, 2016	58–2292	0.7–33.2	7.7 (6.3, 9.3)
Iran (Islamic Republic of Iran)	16	2009, 2010, 2011, 2012, 2013, 2014, 2016	60–1588	0.7–32.4	6.5 (2.7, 11.8)
Maldives	2	2008	129–147	0.0–0.8	0.20 (0.00, 1.40)
Nepal	6	2011, 2015	100–401	0.0–8.0	2.20 (0.60, 4.70)
Pakistan	1	2011	300	43.0	43.00 (37.30, 48.80)
Sub‐Saharan Africa (7 countries)	10	2011–2016	57–620	0.3–10.5	4.5 (2.5, 7.0)
Côte d'Ivoire	1	2016	57	10.5	10.5 (4.0, 21.5)
Kenya	1	2015	371	5.4	5.4 (3.3, 8.2)
Madagascar	3	2012	176–211	3.1–8.2	5.3 (3.6, 7.3)
Mauritius	2	2011	500–511	7.0–9.0	6.3 (4.8, 7.9)
Nigeria	1	2013	328	6.7	6.7 (4.3, 10.0)
Seychelles	1	2011	346	0.3	0.3 (0.0, 1.6)
Tanzania (United Republic of)	1	2014	620	1.1	1.0 (0.4, 2.1)
Western Europe (15 countries)	123	2008–2016	11–2077	0.0–22.5	1.7 (1.4, 2.1)
Austria	5	2016	91–159	2.7–6.6	4.1 (2.7, 5.8)
Belgium	10	2016	32–405	0.0–4.3	1.6 (1.1, 2.3)
Croatia	3	2010	121–150	0.7–1.7	1.0 (0.1, 2.3)
Denmark	1	2010	241	1.3	1.2 (0.3, 3.6)
France	1	2016	908	0.8	0.8 (0.3, 3.6)
Germany	10	2009, 2016	130–2077	0.3–1.5	0.9 (0.6, 1.2)
Greece	70	2016	11–1911	0.0–11.8	1.9 (1.5, 2.2)
Luxembourg	1	2012	310	0.8	0.6 (0.1, 2.3)
Netherlands	6	2016	13–81	0.0–12.5	0.6 (0.0, 4.5)
Norway	4	2009, 2016	116–195	0.9–3.6	2.0 (1.0, 3.3)
Portugal	6	2016	503–1054	2.2–6.8	3.6 (2.1, 5.6)
Serbia	2	2014	199–300	2.8–5.0	3.9 (2.3, 5.8)
Spain	2	2008, 2010	516–1223	1.8–22.5	3.0 (2.2, 3.8)
Sweden	1	2014	277	1.9	0.7 (0.1, 2.6)
United Kingdom	1	2016	2344	1.0	0.9 (0.6, 1.4)
All included countries (55 countries)	304	2008–2016	11–9405	0.0–76.8	4.5 (3.9, 5.1)

Abbreviation: CI, confidence interval.

^a^ These values are unweighted so may differ slightly to the % Infected HBsAg.

### 3.1 Meta‐analysis

**FIGURE 1:**
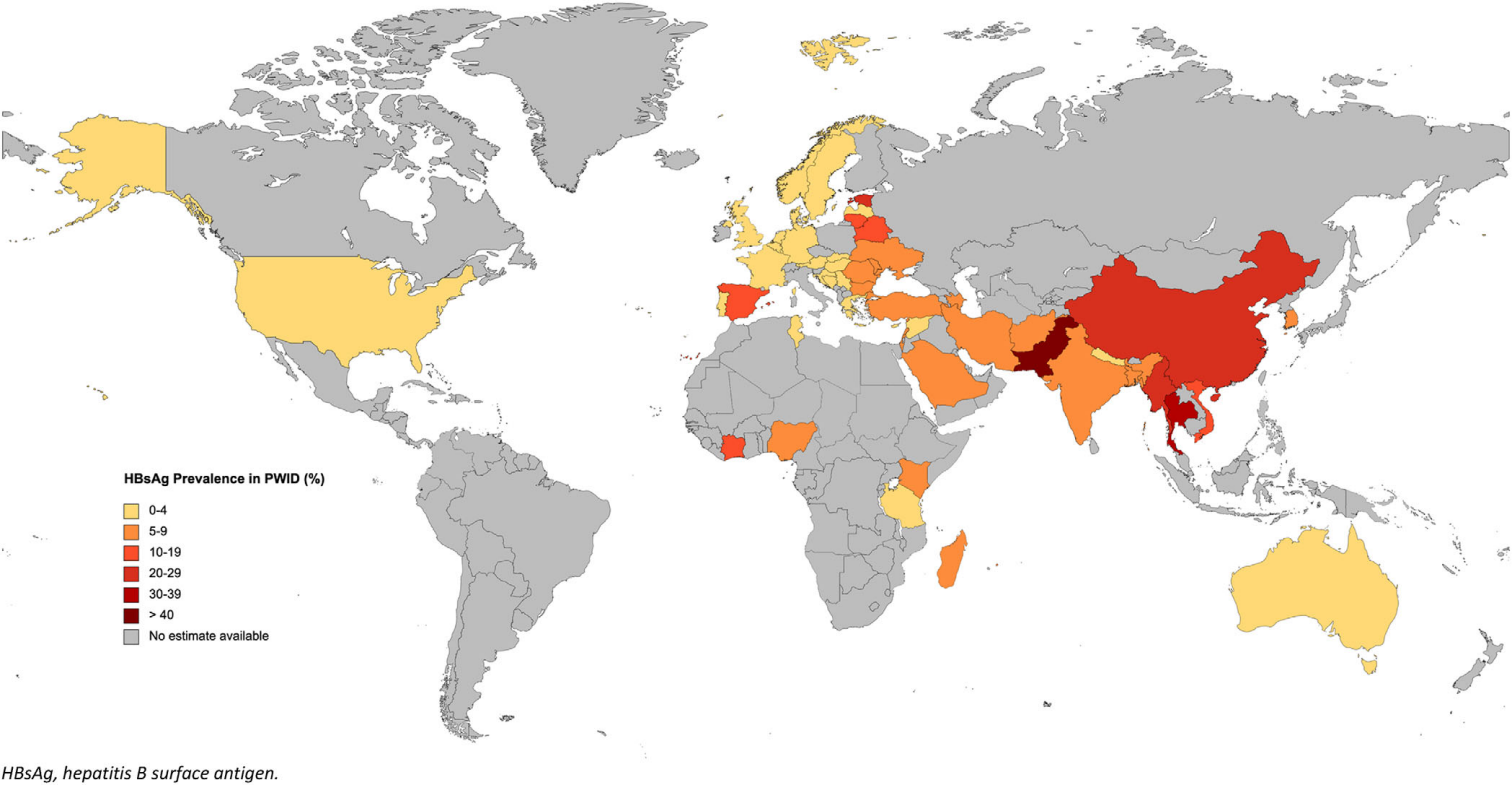
Estimated chronic Hepatitis B virus (HBsAg positive) prevalence in people who inject drugs (PWID). HBsAg, hepatitis B surface antigen

There was marked variation identified in the percentage of PWID infected with HBsAg within estimates, countries and regions. The overall *I* ^2^ statistic value for HBV prevalence among PWID across all estimates was 96.3% (*p* <0.001): indicating considerable to substantial heterogeneity [11] (for individual country‐level *I* ^2^ values, see Table S4). The global mean chronic HBV prevalence in PWID was 4.5% (95% confidence interval [CI] 3.9–5.1) (Table 1; Figure 1). The region with the highest reported percentage of PWID infected with HBsAg was East and Southeast Asia (17.6% [95% CI 13.3–22.3]), and the lowest was Western Europe (1.7% [95% CI 1.4–2.1]) (for regional forest plots see supplementary material pages 13–18). The country with the highest reported mean percentage of PWID infected with HBsAg was Pakistan (43.0 [95% CI 37.3–48.8]—taken from one estimate), and the lowest prevalence was found in Bosnia and Herzegovina (0.2 [95% CI 0.0–1.5]) and the Maldives (0.2 [95% CI 0.0–1.4]).

### 3.2 Ecological analysis

**FIGURE 2:**
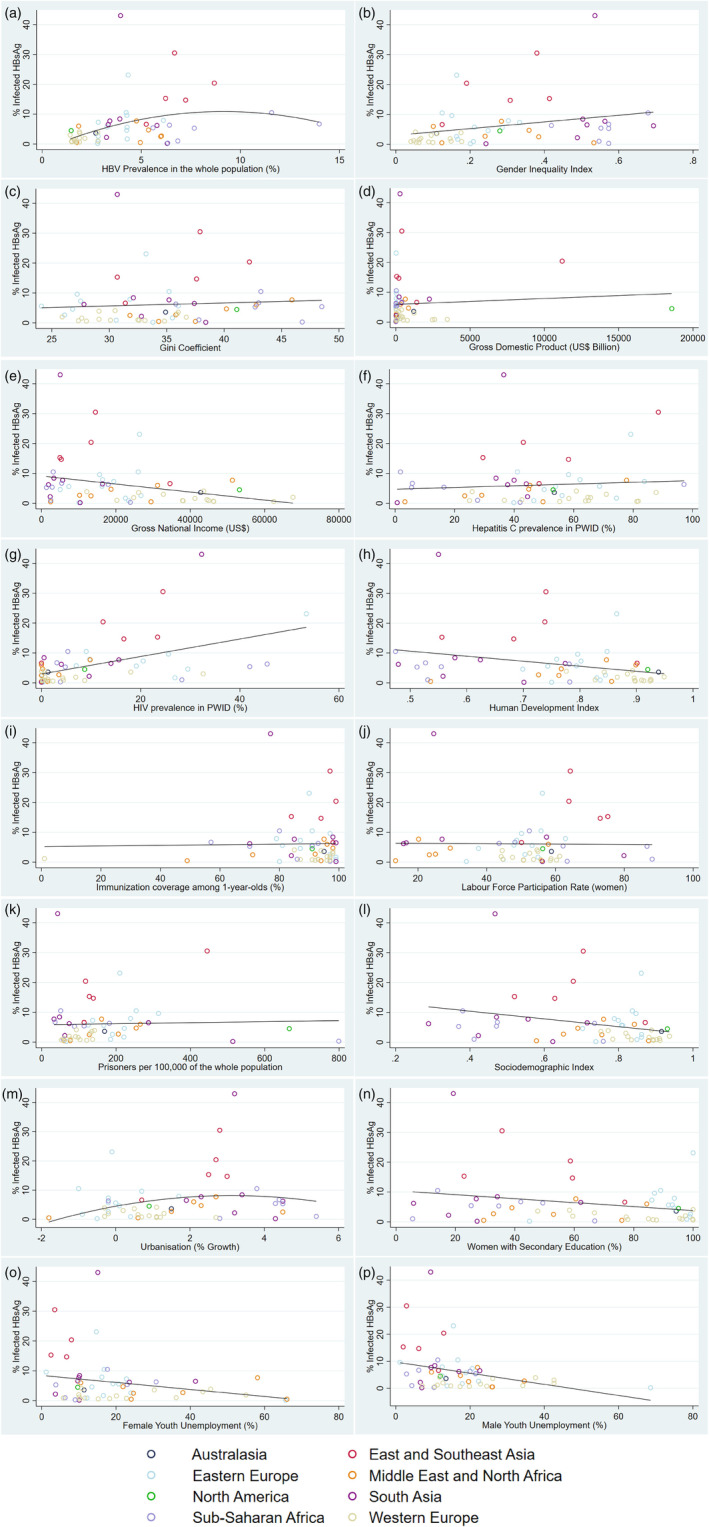
(a–p) Scatter plot for country‐level characteristics and the percentage infected with hepatitis B surface antigen (HBsAg) per country, grouped by UNAIDS region. (a) HBV prevalence in the whole population (%). (b) Gender Inequality Index. (c) Gini Coefficient. (d) Gross domestic product (US$ billion). (e) Gross national income (US$). (f) HCV prevalence in PWID (%). (g) HIV prevalence in PWID (%). (h) Human Development Index. (i) Immunisation coverage among 1‐year‐olds (%). (j) Labour force participation rate (women). (k) Prisoners per 100,00 of the whole population. (l) Sociodemographic Index. (m) Urbanisation (% growth). (n) Women with secondary education (%). (o) Female youth unemployment (%). (p) Male youth unemployment (%). The scatterplots have been superimposed by either a linear or quadratic line of best fit depending on whether the linear or quadratic term had a superior fit in the model. These graphs exhibit a regular scale and have not been logit transformed. HBV, hepatitis B virus; HCV, hepatitis C virus; PWID, people who inject drugs.

For most country‐level characteristics, the linear model demonstrated a superior fit. The model including the quadratic term was deemed a superior fit for the HBV prevalence in the whole population and urbanisation variables (Figure 2a–p). The results from the linear regression are displayed in Table 2.

**TABLE 2:** Univariable and multivariable country‐level linear regression analyses of country‐level characteristics and the prevalence of chronic hepatitis B virus (HBsAg positive) in people who inject drugs (logit transformed dependent variable)

Country‐level characteristic (independent variable)	Univariable model*		Multivariable model**			
	Country‐level independent variable		HBV prevalence among general population		Country‐level independent variable	
	Linear term	Quadratic term	Linear term	Quadratic term	Linear term	Quadratic term
HBV prevalence in the whole population (%)	0.10 (0.02, 0.18); *p* = 0.02	−0.02 (−0.05, 4.20e^−03^); *p* = 0.10				
Gender inequality index	1.53 (−0.40, 3.10); *p* = 0.06	NA	0.50 (0.02, 0.98); *p* = 0.04	−0.03 (−0.06, 3.79e^−03^); *p* = 0.09	−0.49 (−2.97, 2.00); *p* = 0.70	NA
Gini coefficient	0.02 (−0.04, 0.08); *p* = 0.60	NA	0.51 (0.08, 0.94); p = 0.02	−0.03 (−0.05, 2.84e^−03^); *p* = 0.08	−0.03 (−0.09, 0.03); *p* = 0.36	NA
Gross domestic product (US$ billion)	2.61e^−05^ (−6.10e^−05^, 1.13e^−04^); *p* = 0.56	NA	0.48 (0.07, 0.89); *p* = 0.02	−0.03 (−0.06, 4.67e^−03^); *p* = 0.10	5.65e^−05^ (−1.64e^−05^, 1.29e^−04^); *p* = 0.13	NA
Gross National Income (US$)	−2.52e^−05^ (−5.11e^−05^, 7.70e^−07^); *p* = 0.06	NA	0.41 (−0.09, 0.91); *p* = 0.10	−0.02 (−0.05, 0.01); *p* = 0.17	−4.43e^−06^ (−3.60e^−05^, 2.72e^−05^); *p* = 0.78	NA
Human development index	−2.33 (−4.41, −0.25); *p* = 0.03	NA	0.44 (−0.04, 0.92); *p* = 0.07	−0.02 (−0.05, 6.00e^−03^); *p* = 0.12	−0.18 (−3.56, 3.20); *p* = 0.92	NA
Immunisation coverage among 1‐year‐olds (%)	1.27e^−03^ (−0.02, 0.02); *p* = 0.90	NA	0.45 (0.03, 0.87); *p* = 0.04	−0.02 (−0.05, 0.01); *p* = 0.14	0.01 (−0.02, 0.04); *p* = 0.54	NA
Labour force participation rate (women) (%)	−1.21e^−03^ (−0.03, 0.02); *p* = 0.92	NA	0.45 (0.04, 0.87); *p* = 0.03	−0.02 (−0.05, 4.43e^−03^); *p* = 0.10	−4.03e^−04^ (−0.02, 0.02); *p* = 0.96	NA
Prisoners per 100,000 of the whole population	2.28e^−04^ (−1.82e^−03^, 2.29e^−03^); *p* = 0.83	NA	0.48 (0.05, 0.90); *p* = 0.03	−0.03 (−0.06, 4.17e^−03^); *p* = 0.09	−4.11e^−04^ (−2.43e^−03^, 1.61e^−03^); *p* = 0.69	NA
Sociodemographic index	−1.69 (−3.27, −0.10); *p* = 0.04	NA	0.45 (−0.02, 0.93); *p* = 0.06	−0.02 (−0.05, 0.01); *p* = 0.11	0.02 (−2.51, 2.56); *p* = 0.99	NA
Urbanisation (% growth)	0.13 (−0.05, 0.31); *p* = 0.16	−0.66 (−1.60, 0.28); *p* = 0.17	0.48 (0.07, 0.89); *p* = 0.02	−0.02 (−0.05, 4.13e^−03^); *p* = 0.09	0.55 (−0.19, 1.28); *p* = 0.15	−0.13 (−0.27, 0.01); *p* = 0.07
Women with secondary education (%)	−0.01 (−0.02, 3.51e^−04^); *p* = 0.06	NA	0.45 (−0.03, 0.93); *p* = 0.07	−0.02 (−0.05, 0.01); *p* = 0.12	4.81e^−05^ (−0.14, 0.01); *p* = 0.96	NA
Female youth unemployment (%)	−0.03 (−0.07, 0.01); *p* = 0.16	NA	0.42 (0.04, 0.80); *p* = 0.03	−0.02 (−0.05, 4.22e^−03^); *p* = 0.10	−0.02 (−0.06, 0.01); *p* = 0.15	NA
Male youth unemployment (%)	−0.57 (−0.11, −0.01); *p* = 0.03	NA	0.36 (−0.02, 0.74); *p* = 0.06	−0.02 (−0.05, 0.01); *p* = 0.13	−0.04 (−0.08, 3.85e^−03^); *p* = 0.07	NA
HCV prevalence in PWID (%)	0.01 (−0.01, 0.02); *p* = 0.52	NA	0.49 (0.11, 0.87); *p* = 0.01	−0.02 (−0.05, 4.42e^−03^); *p* = 0.10	0.01 (2.82e^−03^, 0.03); *p* = 0.01	NA
HIV prevalence in PWID (%)	0.03 (0.02, 0.05); *p* < 0.001	NA	0.27 (−0.12, 0.65); *p* = 0.17	−0.01 (−0.04, 0.02); *p* = 0.39	0.03 (0.01, 0.04); *p* < 0.001	NA

*Note*: Notation = regression coefficient (95% confidence interval); *p*‐value.

Abbreviations: CI, confidence interval; HBV, hepatitis B virus; HBsAg, hepatitis B surface antigen; HCV, hepatitis C virus; NA, not applicable; PWID, people who inject drugs.

^a^ With country‐level HBV prevalence among PWID as the dependent variable and the country‐level characteristics as independent variables (including a linear and sometimes a quadratic term depending on fit).

^b^ As with the univariable model, but additionally adjusting for country‐level HBV prevalence among the general population.

There was some evidence of an association between HBV prevalence in the whole population and PWID in the univariable model. HBV prevalence in PWID rises as HBV in the general population increases, then begins to fall at the highest levels of HBV prevalence in the general population (Figure 2a). An association between these two variables was also identified in most multivariable models containing other country‐level characteristics when HBV prevalence in the general population was adjusted for.

Some associations between the country‐level characteristics were identified in the univariable model; however, many were attenuated in the multivariable model when adjusting for HBV in the whole population. A weak positive association between Gender Inequality Index and chronic HBV prevalence in PWID was identified (1.53 [95% CI −0.40, 3.10]; *p* = 0.06) but this was attenuated in the multivariable model (−0.49 [95% CI −2.97, 2.00]; *p* = 0.70). There was a negative association between Human Development Index (−2.33 [95% CI −4.41, −0.25]; *p* = 0.03), Gross National Income (−2.52e^−05^ [95% CI −5.11e^−05^, 7.70e^−07^]; *p* = 0.06), Sociodemographic Index (−1.69 [95% CI −3.27, −0.10]; *p* = 0.04), the percentage of women with secondary education (−0.01 [95% CI −0.02, 3.51e^−04^]; *p* = 0.06), and male youth unemployment (−0.57 [95% CI −0.11, −0.01]; *p* = 0.03) and chronic HBV prevalence in PWID. However, these associations were also attenuated (−0.18 [95% CI −3.56, 3.20]; *p* = 0.92), (−4.43e^−06^ [95% CI −3.60e^−05^, 2.72e^−05^]; *p* = 0.78), (0.02 [95% CI −2.51, 2.56]; *p* = 0.99), (4.81e^−05^ [95% CI 0.14, 0.01]; *p* = 0.96) and (−0.04 [95% CI −0.08, 3.85e^−03^]; *p* = 0.07), respectively when adjusting for HBV prevalence in the whole population.

There was no evidence of an association between HCV and chronic HBV prevalence in PWID in the univariable model (0.01 [95% CI −0.01, 0.02]; *p* = 0.52). However, once adjusting for the chronic HBV prevalence in the whole population, there was some evidence of a positive association (0.01 [95% CI 2.82e^−03^, 0.03]; *p* = 0.01). There was evidence of association between HIV and chronic HBV prevalence in the univariable model (0.03 [95% CI 0.02, 0.05]; *p* < 0.001), which was maintained in the multivariable model (0.03 [95% CI 0.01, 0.04]; *p* < 0.001).

There was no evidence of an association between the Gini coefficient, gross domestic product, gross national income, HepB3 (three‐dose) immunisation coverage among 1‐year‐olds, women's labour force participation rate, prison population (per 100,000 people of the population), percentage of women with secondary education, urbanisation or female youth unemployment of a nation and chronic HBV prevalence in PWID, in either the univariable or multivariable model when adjusting for HBV prevalence in the whole population.

## 4 DISCUSSION

We found that HCV and HIV prevalence in PWID were positively associated with chronic HBV prevalence among PWID in multivariable analyses. There was some evidence also of associations between country‐level measures of social disadvantage and chronic HBV prevalence in PWID; including negative associations with Human Development Index score, Gross National Income, Sociodemographic Index score, the percentage of women with secondary education, levels of male youth unemployment and a positive association with Gender Inequality Index score. The general rule of these univariable results was that higher development or less inequality was associated with lower HBV prevalence. The exception to this was the negative association between male youth unemployment and HBV, which was perhaps acting as a marker of country‐income as, for example, male youth unemployment was 22.37% on average in high‐income Western European countries that have low HBV prevalence, while these figures were 10.95% in sub‐Saharan Africa and 11.80% in South Asia, which contained high HBV prevalence low‐ and middle‐income countries. However, these associations of country‐level measures of social disadvantage with HBV prevalence among PWID were all attenuated after adjusting for prevalence of HBV in the whole population, which did not corroborate our hypothesis that countries with higher levels of inequality and poverty would have higher HBV prevalence among PWID when controlling through general population HBV prevalence. Thus, there is a clear rationale for investing in comprehensive public health policies to reduce HBV in the whole population, not just in PWID [1], reinforced by the inclusion of combatting HBV as one of the United Nation's Sustainable Development Goals [12]. Our study highlights that HBV is an ongoing and compared to other comorbidities, often neglected (due to the quantity of countries missing data on HBV but not on HCV or HIV), issue among PWID, that is particularly problematic in East and Southeast Asia.

### 4.1 Other evidence

The positive association between HCV and HIV prevalence in PWID and chronic HBV prevalence in PWID was expected due to the high co‐infection rates of bloodborne viruses among PWID [13, 14, 15], highlighting the need for interventions targeted at PWID to address multiple health harms. Evidence on drug related harm has highlighted the need for improving harm reduction services [4], including introducing HBV vaccination for adults in high‐risk groups such as PWID [3]. It has been suggested that less than one in three PWID have completed the vaccination series as adults [4]. As a result of exposure to increased high‐risk environments and collective stigmatisation towards drug use, PWID are one of the most socially and medically vulnerable populations [16]. These disparities in health‐care equality catalyse an environment where the help‐seeking behaviours of PWID are impacted, often making PWID reluctant to engage with clinical services, like vaccine uptake, increasing their susceptibility to HBV [17].

HBV elimination efforts have been described as ‘successful but fragmented’ by the World Health Organization. Low‐income countries traditionally lack the financial resources and health infrastructure to implement effective prevention, such as universal vaccine programs or have only developed the propensity recently. These countries often suffer the heaviest burden of HBV and score lower on multiple indices capturing socioeconomic and development [18], as indicated by the associations identified in our univariable analyses between HBV prevalence among PWID and various development indices. A comprehensive disease management strategy requires both high level commitment, perhaps even legislation, and funding to increase health promotion, screening, vaccination and treatment [9]. A global investigation into the health governance of HBV and country‐level socioeconomic factors found that countries were more likely to have routine viral hepatitis surveillance and a national strategy for preventing infection if they were in the higher binary categories for income level and health expenditure, all traditionally associated with high‐income countries [19].

Some countries are still struggling to implement universal vaccination programs in hyperendemic rural areas, but, for the most part, coverage continues to increase [3, 18]. However, the roll‐out of HBV vaccine in the 1990s means that a sizeable proportion of PWID may not have been inoculated as infants; the median age for PWID in this analysis was 31.4 years. While we did not find an association between immunisation coverage and HBV prevalence in this analysis, repeating this analysis in 5 to 10 years, when the effects of previous childhood vaccination will have wider impact would be a further test of health inequalities. High vaccination rates will have a powerful impact on eradicating HBV in the coming decades, however, there is a need to improve the understanding of the associations between financial resources and the epidemiology of HBV infection in PWID, to design and implement cost‐effective public health interventions [1]. Examples include catch‐up vaccination programs for key populations, harm reduction interventions like needle and syringe programs and opioid substitution therapy and improving screening, testing and antiviral therapy. The latter constitutes a substantial financial burden and acts as a limiting factor in HBV infection management in low‐ and middle‐income countries [20].

### 4.2 Strengths and limitations

To the best of our knowledge, this is the first ecological analysis exploring the association between country‐level chronic HBV prevalence in PWID and factors of development. One limitation of our study was that data were incomplete for some country‐level characteristics, so, some values were imputed. Furthermore, as the exposures were measured at the national level rather than the individual level, the interpretation of these results could be subject to an ecological fallacy and the association observed at a country/national level not hold for an individual [21]. For example, at a country‐level, the number of prisoners per 100,000 of the whole population was not associated with the country‐level HBV prevalence among PWID, perhaps because this variable may not adequately summarise differing prison‐based exposure risks for HBV across countries. However, at an individual‐level, PWID who have been imprisoned may have higher odds of having HBV due to exposures such as prison tattoos or a lack of clean needles and syringes being available in prisons [22]. There also was extensive missing data from many countries, due to the absence of studies on PWID and HBsAg, with only 55 of 179 countries included of those with reported evidence of injecting drug use [5]. Data were unavailable for Latin America, the Caribbean and the Western Pacific, as well as large expanses of sub‐Saharan Africa (Figure 1), regions where HBV is endemically high [23]. Furthermore, estimates for 20 countries comprised of only one study, which limits confidence in generalisability, and ultimately the external validity of the results. Finally, limited data on exposure to injecting drug use and risk of bloodborne viruses in PWID generates uncertainty in the estimates [8, 13]. To emphasise this uncertainty, we note that the estimate we used for Pakistan (43.0 [95% CI 37.3, 48.8]) was substantially higher than the 22.4% [24] and 7.5% [25] found by other studies.

### 4.3 Implications

HBV is an ongoing issue among PWID, that is often neglected compared with other viral diseases. This is evidenced by the substantial amount of missing data on HBV in countries with data on HIV and HCV, and well‐developed strategies in many countries to eliminate HCV and HIV, which do not always apply to HBV. The association of HBV prevalence among PWID with the HBV prevalence among the general population shows that there is also a need for investing in comprehensive public health policies to reduce HBV in the whole population, as well as targeting vulnerable populations such as PWID. It is too early to assess whether expanding childhood vaccination will also reduce chronic HBV in PWID populations. Our review also emphasises the need for increased transparent epidemiological surveillance of HBV prevalence in PWID to help establish effective strategies for harm reduction, particularly in countries where HBV is endemic [10, 26].

## AUTHOR CONTRIBUTIONS

Anjalee Syangbo conducted the analysis, generated the estimates, and drafted the first iteration of the manuscript. Adam Trickey, Matthew Hickman and Anjalee Syangbo conceived and designed the present study. Adam Trickey, Samantha Colledge‐Frisby, Janni Leung, Jason Grebely, Sarah Larney, Louisa Degenhardt, and Matthew Hickman made substantial contributions to the acquisition of data. Adam Trickey, Matthew Hickman and Anjalee Syangbo contributed to the study methods and analysis plan. All authors contributed to revising the manuscript critically for important intellectual content.

## FUNDING INFORMATION

This research was funded in whole, or in part, by the Wellcome Trust 222770/Z/21/Z. For the purpose of Open Access, the author has applied a CC BY public copyright licence to any Author Accepted Manuscript version arising from this submission. This project was supported with funding from the Australian National Drug and Alcohol Research Center, UNSW Sydney; Open Society Foundation; World Health Organization; Global Fund; and UNAIDS. Janni Leung and Louisa Degenhardt are supported by Australian National Health and Medical Research Council Fellowships. Jason Grebely is supported by an Australian National Health and Medical Research Council Investigator Grant. Sarah Larney is supported by a Research Scholar award from Fonds de recherche du Québec—Santé. Janni Leung acknowledges funding from the Bill & Melinda Gates Foundation. Matthew Hickman acknowledges support from the National Institute for Health and Care Research Health Protection Research Unit in Behavioural Science and Evaluation. Samantha Colledge‐Frisby acknowledges funding from the UNSW Scientia PhD Scholarship scheme. The National Drug and Alcohol Research Centre, the Kirby Institute and the National Centre for Youth Substance Use Research are funded by the Australian Government Department of Health and Ageing. The views expressed in this publication do not necessarily represent the position of the Australian Government. Funding sources had no role in the study design; collection, analysis and interpretation of data; writing of the report; or decision to submit the article for publication.

## CONFLICT OF INTEREST

Sarah Larney has received investigator‐initiated untied educational grants from Indivior. Louisa Degenhardt has received investigator‐initiated untied educational grants for studies of opioid medications in Australia from Reckitt Benckiser, Indivior, Mundipharma and Seqirus. Matthew Hickman reports honoraria for speaking at meetings from Gilead, Abbvie, and MSD. Jason Grebely is a consultant and adviser for and has received research grants from Abbvie, Camurus, Cepheid, Gilead Sciences, Hologic, Indivior and Merck/MSD.

## Supporting information


**Appendix S1.** Supporting Information.
