# Supplementary material for: Associations between the prevalence of chronic hepatitis B among people who inject drugs and country‐level characteristics: An ecological analysis
Source: Drug Alcohol Rev. 2023 Jan 4;42(3):569–81. doi: 10.1111/dar.13595 (PMC10728688; doi:10.1111/dar.13595)
Supplement: Supplementary file 1 — Appendix S1. Supporting Information. [file DAR-42-569-s001.docx]

**Supplementary Materials**

# **Figure S1.** Study flow diagram for systematic review of the prevalence of injecting drug use and HIV, hepatitis C and hepatitis B among people who inject drugs* from which the data on hepatitis B were taken.


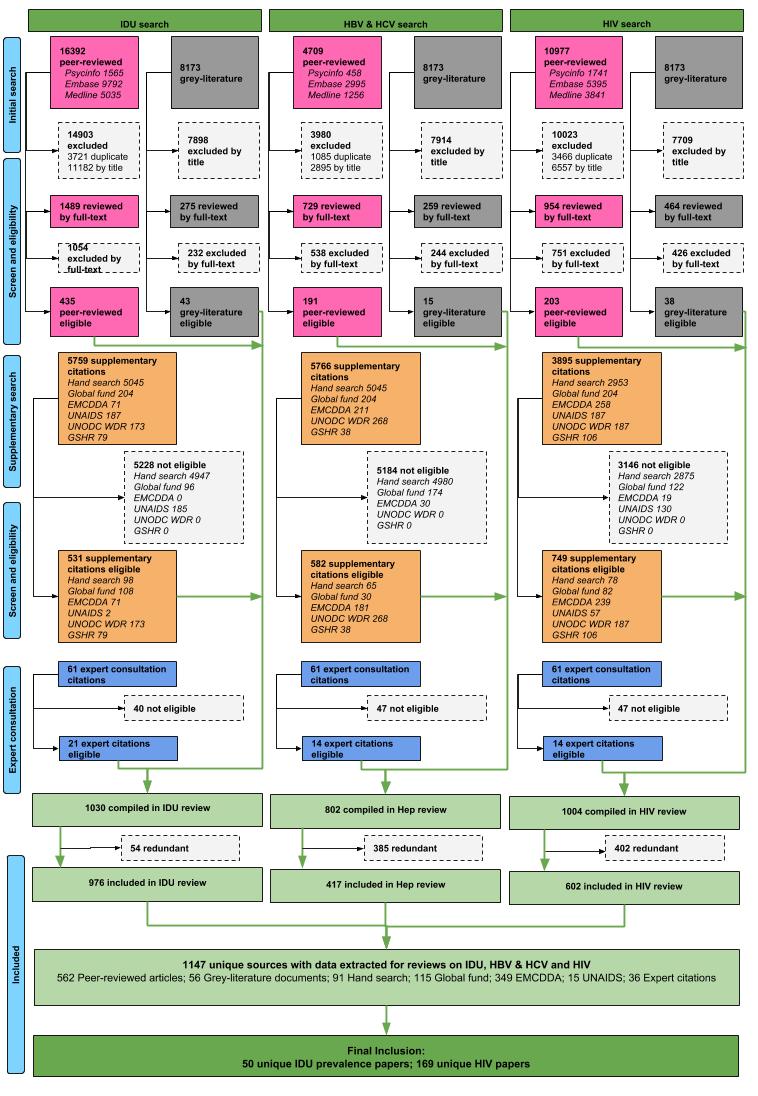


*Taken from Degenhardt L, Peacock A, Colledge S, Leung J, Grebely J, Vickerman P, et al. Global prevalence of injecting drug use and sociodemographic characteristics and prevalence of HIV, HBV, and HCV in people who inject drugs: a multistage systematic review. Lancet Glob Health. 2017;5:e1192-e207.

# **Table S1.** GATHER checklist.

| Item # | Checklist item | Reported on page # |
| --- | --- | --- |
| Objectives and funding | | |
| 1 | Define the indicator(s), populations (including age, sex, and geographic entities), and time period(s) for which estimates were made. | 4 |
| 2 | List the funding sources for the work. | 1 |
| Data inputs | | |
| *For all data inputs from multiple sources that are synthesized as part of the study:* | | |
| 3 | Describe how the data were identified and how the data were accessed. | 4 |
| 4 | Specify the inclusion and exclusion criteria. Identify all ad-hoc exclusions. | Supplement page 2 |
| 5 | Provide information on all included data sources and their main characteristics. For each data source used, report reference information or contact name/institution, population represented, data collection method, year(s) of data collection, sex and age range, diagnostic criteria or measurement method, and sample size, as relevant. | 4, 12-14; Supplement pages 4-11 |
| 6 | Identify and describe any categories of input data that have potentially important biases (e.g., based on characteristics listed in item 5). | NA |
| *For data inputs that contribute to the analysis but were not synthesized as part of the study:* | | |
| 7 | Describe and give sources for any other data inputs. | 4, 12-14; Supplement pages 4-11 |
| *For all data inputs:* | | |
| 8 | Provide all data inputs in a file format from which data can be efficiently extracted (e.g., a spreadsheet rather than a PDF), including all relevant meta-data listed in item 5. For any data inputs that cannot be shared because of ethical or legal reasons, such as third-party ownership, provide a contact name or the name of the institution that retains the right to the data. | Table 1 and supplement table 3 contain all data included in ecological analyses |
| Data analysis | | |
| 9 | Provide a conceptual overview of the data analysis method. A diagram may be helpful. | 4-5 |
| 10 | Provide a detailed description of all steps of the analysis, including mathematical formulae. This description should cover, as relevant, data cleaning, data pre-processing, data adjustments and weighting of data sources, and mathematical or statistical model(s). | 4-5 |
| 11 | Describe how candidate models were evaluated and how the final model(s) were selected. | 4-5 |
| 12 | Provide the results of an evaluation of model performance, if done, as well as the results of any relevant sensitivity analysis. | 6 |
| 13 | Describe methods for calculating uncertainty of the estimates. State which sources of uncertainty were, and were not, accounted for in the uncertainty analysis. | 4-5 |
| 14 | State how analytic or statistical source code used to generate estimates can be accessed. | 5 |
| Results and discussion | | |
| 15 | Provide published estimates in a file format from which data can be efficiently extracted. | 12-14 |
| 16 | Report a quantitative measure of the uncertainty of the estimates (e.g. uncertainty intervals). | 12-14 |
| 17 | Interpret results in light of existing evidence. If updating a previous set of estimates, describe the reasons for changes in estimates. | 6-8 |
| 18 | Discuss limitations of the estimates. Include a discussion of any modelling assumptions or data limitations that affect interpretation of the estimates. | 9 |

Page numbers refer to the submitted manuscript.

**Table S2.** Further details of sources country-level characteristics

| **Indicator (year)** | **Definition** | **Additional notes** |
| --- | --- | --- |
| HBV prevalence within the whole population (%) (2015) | The percentage of the whole population of a country infected with hepatitis B virus (HBV) |  |
| Gender Inequality Index (2014) | Reflects gender-based disadvantage in three dimensions: reproductive health, empowerment, and the labour market.  *Scale:*  *0 = men and women fare equally*  *1 = one gender fares as poorly as possible in all dimensions* [1] | Data for Nigeria, Madagascar, Seychelles were imputed using the Sub-Saharan African regional average (2020) [2] |
| Gini Coefficient (2015) | A statistical measure of distribution intended to represent the income or wealth distribution (income inequality).  *Scale:*  *0 = perfect equality*  *1 = perfect inequality* [3] | Data for Korea (2015), Myanmar (2017), Lebanon (2011), Saudi Arabia (2013), the Syrian Arab Republic (2003) and Afghanistan (2007) from the most recent available year were obtained [3] |
| Gross domestic product (US$ billion) (2016) | The total monetary or market value of all the finished goods and services produced within a country's borders in a specific time period. Functions as a comprehensive scorecard of a country's economic health [4] | Data for the Syrian Arab Republic was obtained from the most recent year (2019) [5] |
| Gross national income (US$) (2016) | The total amount of money earned by a nation's people and businesses. It is used to measure and track a nation's wealth from year to year. Includes the nation’s gross domestic product plus the income it receives from overseas sources [6] |  |
| Hepatitis C prevalence in PWID (%) | Percentage of PWID with hepatitis C (per country) |  |
| HIV prevalence in PWID (%) | Percentage of PWID with HIV (per country) |  |
| Human Development Index (2016) | A summary measure of average achievement in key dimensions of human development: a long and healthy life, being knowledgeable and have a decent standard of living.  *Scale:*  *≥ 0.800 = very high human development*  *0.700-0.799 = high human development*  *0.550-0.699 = medium human development*  *≤ 0.550 = low human development* [1] |  |
| Immunisation coverage among 1-year-olds (%) (2019/2020) | Percentage of 1-year-old infants for that year that have received their complete HepB3 vaccination series | Data was obtained from the most recent year available (2019-2020). As of 2021, Denmark does not have a universal immunisation program and only vaccinate high-risk groups. Therefore the estimated coverage was imputed as 1% [7] |
| Labour Force Participation Rate (women) (%) (2013) | A measure of an economy's female active workforce. The sum of all women workers who are employed or actively seeking employment divided by the total civilian working-age population [8] | Data for Seychelles was obtained from the most recent year (2019) [9] |
| Prisoners per 100,000 of the whole population (2017) | The number of people incarcerated for every 100,000 people of the whole population |  |
| Sociodemographic Index (2015) | A composite indicator of development status that is strongly correlated with health outcomes. It is the geometric mean total fertility rate under 25, mean education for those aged 15 and older, and the lag distributed income per capita.  *Scale:*  *0 = theoretical minimum level of development relevant to health*  *1 = theoretical maximum level of development relevant to health* [10] |  |
| Urbanisation (% Groh) (2015) | A measure of how fast the population is urbanising (when the number of people living in urban areas is increasing) |  |
| Women with secondary education (%) (2014) | Percentage of women in the population who have completed secondary education | Data for Madagascar and Nigeria was obtained from the most recent year (2020-2021) [11,12] |
| Female youth unemployment (%) (2016) | The percentage of young women aged 15-24 that are unemployed | Data for Seychelles was obtained for the most recent year (2019) [13] |
| Male youth unemployment (%) (2016) | The percentage of young men aged 15-24 that are unemployed | Data for Seychelles was obtained for the most recent year (2019) [14] |

This data was obtained from online databases such as the World Bank Catalogue^(15)^, the United Nations Development Programme's Human Development Reports^(2)^, The Global Burden of Disease study^(16)^ and the World Health Organisation's Global Health Observatory^(17)^.

PWID, people who inject drugs

**Table S3.** Country-level socioeconomic, development and health characteristic values (grouped by UNAIDS region)

| **Country of report** | **HBV prevalence in the whole population (%)** | **Gender Inequality Index** | **Gini Coefficient** | **Gross domestic product (US$ Billion)** | **Gross national income (US$)** | **Hepatitis C prevalence in PWID (%)** | **HIV prevalence in PWID (%)** | **Human Development Index** | **Immunisation coverage among 1-year-olds (%)** | **Labour force participation rate (women) (%)** | **Prisoners per 100,000 of the whole population** | **Sociodemographic Index** | **Urbanisation (% growth)** | **Women with secondary education (%)** | **Female Youth unemployment (%)** | **Male Youth unemployment (%)** |
| --- | --- | --- | --- | --- | --- | --- | --- | --- | --- | --- | --- | --- | --- | --- | --- | --- |
| **Australasia** | | | | | | | | | | | | | | | | |
| Australia | 2.71 | 0.11 | 34.90 | 1204.60 | 42822 | 53.50 | 1.30 | 0.94 | 95 | 58.80 | 169 | 0.92 | 1.50 | 94.30 | 11.40 | 13.50 |
| **Average** | **2.71** | **0.11** | **34.90** | **1204.60** | **42822.00** | **53.50** | **1.30** | **0.94** | **95** | **58.80** | **169.00** | **0.92** | **1.50** | **94.30** | **11.40** | **13.50** |
| **East and Southeast Asia** | | | | | | | | | | | | | | | | |
| China | 8.68 | 0.19 | 42.20 | 11199.10 | 13345 | 43.10 | 12.40 | 0.74 | 99 | 63.90 | 118 | 0.68 | 2.70 | 58.70 | 8.00 | 12.90 |
| Korea (Republic of) | 5.25 | 0.12 | *31.40* | 1411.20 | 34541 | 48.40 | 0.00 | 0.90 | 98 | 50.10 | 114 | 0.87 | 0.70 | 77.00 | 9.70 | 11.50 |
| Myanmar | 6.23 | 0.41 | *30.70* | 67.40 | 4943 | 29.50 | 23.40 | 0.56 | 84 | 75.20 | 128 | 0.52 | 2.50 | 22.90 | 2.50 | 2.00 |
| Thailand | 6.68 | 0.38 | 37.90 | 406.80 | 14519 | 88.50 | 24.50 | 0.74 | 97 | 64.30 | 445 | 0.70 | 2.80 | 35.70 | 3.50 | 2.90 |
| Vietnam | 7.24 | 0.31 | 37.60 | 202.60 | 5335 | 58.30 | 16.60 | 0.68 | 94 | 73.00 | 139 | 0.63 | 3.00 | 59.40 | 6.70 | 6.20 |
| **Average** | **6.82** | **0.28** | **35.96** | **2657.42** | **14536.60** | **53.56** | **15.38** | **0.72** | **94.40** | **65.30** | **188.80** | **0.68** | **2.34** | **50.74** | **6.08** | **7.10** |
| **Eastern Europe** | | | | | | | | | | | | | | | | |
| Azerbaijan | 6.12 | 0.30 | 31.80 | 37.80 | 16413 | 62.10 | 9.70 | 0.76 | 79 | 62.90 | 239 | 0.79 | 1.70 | 93.70 | 16.20 | 12.50 |
| Belarus | 4.27 | 0.15 | 27.20 | 47.40 | 15629 | 58.30 | 25.60 | 0.80 | 97 | 50.10 | 314 | 0.85 | 0.70 | 87.00 | 1.20 | 1.10 |
| Bosnia and Herzegovina | 2.80 | 0.20 | 33.80 | 16.60 | 10091 | 39.90 | 0.30 | 0.75 | 80 | 34.10 | 66 | 0.74 | -0.50 | 44.90 | 65.70 | 68.60 |
| Bulgaria | 3.28 | 0.21 | 36.00 | 52.40 | 16261 | 68.70 | 7.00 | 0.79 | 91 | 47.90 | 125 | 0.81 | -0.20 | 93.00 | 19.20 | 18.90 |
| Estonia | 4.34 | 0.16 | 33.20 | 23.10 | 26362 | 79.20 | 53.40 | 0.87 | 90 | 56.20 | 210 | 0.86 | -0.10 | 100.00 | 14.70 | 15.50 |
| Hungary | 2.83 | 0.21 | 30.60 | 124.30 | 23394 | 46.60 | 0.20 | 0.84 | 99 | 44.80 | 185 | 0.85 | 0.40 | 97.90 | 12.20 | 13.80 |
| Latvia | 4.29 | 0.17 | 35.50 | 27.70 | 22589 | 74.40 | 26.90 | 0.83 | 99 | 54.90 | 221 | 0.86 | -0.90 | 98.90 | 16.60 | 17.90 |
| Lithuania | 4.24 | 0.12 | 35.20 | 42.70 | 26006 | 41.10 | 9.00 | 0.85 | 91 | 55.80 | 254 | 0.84 | -1.00 | 89.10 | 16.80 | 16.70 |
| Moldova (Republic of) | 4.26 | 0.25 | 26.80 | 6.70 | 5026 | 50.10 | 29.50 | 0.70 | 87 | 37.60 | 222 | 0.70 | 0.10 | 93.60 | 13.50 | 12.50 |
| Romania | 2.83 | 0.33 | 27.50 | 186.70 | 19428 | 83.80 | 20.50 | 0.80 | 87 | 48.70 | 137 | 0.80 | -0.20 | 86.10 | 22.80 | 20.60 |
| Slovakia | 2.82 | 0.16 | 26.10 | 89.60 | 26764 | 56.10 | 0.10 | 0.85 | 97 | 51.10 | 190 | 0.86 | -0.20 | 99.10 | 24.00 | 22.30 |
| Ukraine | 4.28 | 0.29 | 24.10 | 93.30 | 7361 | 53.90 | 19.10 | 0.74 | 81 | 53.20 | 167 | 0.81 | 0.00 | 91.70 | 20.70 | 21.70 |
| **Average** | **3.86** | **0.21** | **30.65** | **62.36** | **17943.67** | **59.52** | **16.78** | **0.80** | **89.83** | **49.78** | **194.17** | **0.81** | **-0.02** | **89.58** | **20.30** | **20.18** |
| **Middle East and North Africa** | | | | | | | | | | | | | | | | |
| Cyprus | 1.74 | 0.12 | 34.30 | 19.80 | 29459 | 49.70 | 1.20 | 0.86 | 94 | 56.00 | 77 | 0.88 | 0.60 | 76.00 | 24.10 | 26.00 |
| Israel | 1.84 | 0.10 | 42.80 | 318.70 | 31215 | 45.30 | 0.00 | 0.90 | 96 | 57.90 | 265 | 0.84 | 2.10 | 84.40 | 10.50 | 9.60 |
| Lebanon | 5.98 | 0.38 | *31.80* | 47.50 | 13312 | 23.40 | 0.00 | 0.76 | 71 | 23.30 | 128 | 0.75 | 4.50 | 53.00 | 24.70 | 19.60 |
| Saudi Arabia | 4.76 | 0.28 | *45.90* | 646.40 | 51320 | 77.80 | 9.80 | 0.85 | 95 | 20.20 | 161 | 0.76 | 2.70 | 60.50 | 58.10 | 22.00 |
| Syrian Arab Republic | 4.95 | 0.53 | *37.50* | *27.26* | 2441 | 3.30 | 0.00 | 0.54 | 49 | 13.50 | 60 | 0.58 | -1.80 | 29.50 | 66.00 | 25.90 |
| Tunisia | 6.02 | 0.24 | 35.80 | 42.10 | 10249 | 29.10 | 3.50 | 0.73 | 92 | 25.10 | 206 | 0.65 | 1.50 | 32.80 | 38.00 | 34.60 |
| Turkey | 5.36 | 0.36 | 40.20 | 857.70 | 18705 | 44.90 | 0.20 | 0.77 | 98 | 29.40 | 254 | 0.69 | 2.30 | 39.00 | 21.90 | 17.40 |
| **Average** | **4.38** | **0.29** | **38.33** | **279.92** | **22385.86** | **39.07** | **2.10** | **0.77** | **85** | **32.20** | **164.43** | **0.74** | **1.70** | **53.60** | **34.76** | **22.16** |
| **North America** | | | | | | | | | | | | | | | | |
| United States of America | 1.47 | 0.28 | 41.10 | 18569.10 | 53245 | 53.10 | 8.70 | 0.92 | 91 | 56.30 | 666 | 0.93 | 0.90 | 95.10 | 9.70 | 12.00 |
| **Average** | **1.47** | **0.28** | **41.10** | **18569.10** | **53245.00** | **53.10** | **8.70** | **0.92** | **91** | **56.30** | **666.00** | **0.93** | **0.90** | **95.10** | **9.70** | **12.00** |
| **South Asia** | | | | | | | | | | | | | | | | |
| Afghanistan | 5.79 | 0.69 | *27.80* | 19.50 | 1871 | 37.80 | 4.00 | 0.48 | 70 | 15.80 | 74 | 0.29 | 4.50 | 5.90 | 23.60 | 17.00 |
| Bangladesh | 3.92 | 0.50 | 32.10 | 221.40 | 3341 | 33.90 | 0.50 | 0.58 | 98 | 57.40 | 48 | 0.47 | 3.40 | 34.10 | 10.20 | 10.50 |
| India | 3.41 | 0.56 | 35.20 | 2263.50 | 5663 | 40.00 | 15.60 | 0.62 | 85 | 27.00 | 33 | 0.56 | 2.30 | 27.00 | 10.00 | 9.50 |
| Iran (Islamic Republic of Iran) | 3.33 | 0.51 | 37.40 | 393.40 | 16395 | 44.10 | 14.00 | 0.77 | 99 | 16.60 | 287 | 0.72 | 1.90 | 62.20 | 41.40 | 22.60 |
| Maldives | 6.31 | 0.24 | 38.40 | 3.60 | 10383 | 0.70 | 0.00 | 0.70 | 99 | 56.20 | 514 | 0.62 | 4.30 | 27.30 | 10.10 | 7.00 |
| Nepal | 3.24 | 0.49 | 32.80 | 21.10 | 2337 | 44.50 | 9.60 | 0.56 | 84 | 79.90 | 62 | 0.42 | 3.20 | 17.70 | 3.70 | 6.60 |
| Pakistan | 3.96 | 0.54 | 30.70 | 283.70 | 5031 | 36.50 | 32.30 | 0.55 | 77 | 24.60 | 43 | 0.47 | 3.20 | 19.30 | 15.10 | 9.40 |
| **Average** | **4.28** | **0.50** | **33.49** | **458.03** | **6431.57** | **33.93** | **10.86** | **0.61** | **87.43** | **39.64** | **151.57** | **0.51** | **3.26** | **27.64** | **16.30** | **11.80** |
| **Sub-Saharan Africa** | | | | | | | | | | | | | | | | |
| Cote d'Ivoire | 11.58 | 0.68 | 43.20 | 36.20 | 3163 | 1.80 | 5.30 | 0.47 | 80 | 52.40 | 51 | 0.38 | 3.80 | 14.00 | 17.70 | 11.30 |
| Kenya | 5.59 | 0.55 | 48.50 | 70.50 | 2881 | 16.40 | 42.00 | 0.56 | 91 | 62.20 | 114 | 0.47 | 4.30 | 25.30 | 23.00 | 21.50 |
| Madagascar | 7.67 | *0.57* | 42.70 | 10.00 | 1320 | 5.50 | 4.80 | 0.51 | 70 | 86.60 | 88 | 0.37 | 4.50 | *34.60* | 3.80 | 2.90 |
| Mauritius | 6.42 | 0.42 | 35.80 | 12.20 | 17948 | 97.10 | 45.50 | 0.78 | 93 | 43.60 | 182 | 0.74 | -0.20 | 49.40 | 30.80 | 20.00 |
| Nigeria | 13.98 | *0.57* | 43.00 | 405.10 | 5443 | 5.80 | 3.10 | 0.53 | 57 | 48.20 | 36 | 0.47 | 4.40 | *42.00* | 9.80 | 6.20 |
| Seychelles | 6.34 | *0.57* | 46.80 | 1.40 | 23886 | 42.00 | 3.80 | 0.78 | 97 | *63.40* | 799 | 0.76 | 2.80 | 66.90 | *9.00* | *10.30* |
| Tanzania (United Republic of) | 6.84 | 0.55 | 37.80 | 47.40 | 2467 | 27.70 | 28.30 | 0.53 | 86 | 88.10 | 58 | 0.41 | 5.40 | 5.60 | 6.30 | 4.30 |
| **Average** | **8.35** | **0.56** | **42.54** | **83.26** | **8158.29** | **28.04** | **18.97** | **0.59** | **82.00** | **63.50** | **189.71** | **0.51** | **3.57** | **33.97** | **14.34** | **10.93** |
| **Western Europe** | | | | | | | | | | | | | | | | |
| Austria | 1.88 | 0.05 | 30.50 | 386.40 | 43609 | 60.90 | 0.60 | 0.89 | 85 | 54.60 | 93 | 0.89 | 1.10 | 100.00 | 10.80 | 11.80 |
| Belgium | 1.62 | 0.06 | 27.60 | 466.40 | 41243 | 58.40 | 4.30 | 0.90 | 97 | 47.50 | 98 | 0.88 | 0.60 | 77.50 | 20.10 | 22.80 |
| Croatia | 2.81 | 0.15 | 32.50 | 50.40 | 20291 | 36.70 | 0.40 | 0.83 | 93 | 44.70 | 78 | 0.78 | -0.30 | 85.00 | 36.20 | 34.20 |
| Denmark | 1.64 | 0.05 | 29.10 | 306.10 | 44519 | 42.60 | 1.30 | 0.93 | *1* | 58.70 | 59 | 0.91 | 0.90 | 95.50 | 10.10 | 11.20 |
| France | 1.67 | 0.09 | 33.10 | 2465.50 | 38085 | 64.00 | 8.70 | 0.90 | 91 | 50.70 | 103 | 0.83 | 0.70 | 78.00 | 22.80 | 24.50 |
| Germany | 1.48 | 0.04 | 30.10 | 3466.80 | 45000 | 65.00 | 4.40 | 0.93 | 87 | 53.60 | 77 | 0.90 | 1.10 | 96.30 | 5.80 | 7.10 |
| Greece | 2.15 | 0.15 | 36.70 | 194.60 | 24808 | 65.70 | 6.90 | 0.87 | 96 | 44.20 | 91 | 0.82 | -0.20 | 59.50 | 55.00 | 42.60 |
| Luxembourg | 1.88 | 0.10 | 34.80 | 59.90 | 62471 | 81.30 | 1.50 | 0.90 | 96 | 50.70 | 121 | 0.91 | 2.70 | 100.00 | 15.20 | 17.30 |
| Netherlands | 1.71 | 0.06 | 28.00 | 770.80 | 46326 | 55.30 | 2.30 | 0.92 | 92 | 58.50 | 61 | 0.89 | 1.10 | 87.70 | 10.30 | 10.50 |
| Norway | 1.89 | 0.07 | 25.90 | 370.60 | 67614 | 64.80 | 0.70 | 0.95 | 97 | 61.20 | 74 | 0.94 | 1.30 | 97.40 | 10.00 | 12.70 |
| Portugal | 1.90 | 0.11 | 36.00 | 204.60 | 26104 | 87.70 | 18.00 | 0.84 | 98 | 54.90 | 135 | 0.75 | 0.50 | 47.70 | 30.30 | 26.10 |
| Serbia | 2.81 | 0.18 | 29.10 | 37.70 | 12202 | 25.90 | 0.00 | 0.78 | 94 | 44.50 | 142 | 0.77 | -0.30 | 58.40 | 45.50 | 37.60 |
| Spain | 1.41 | 0.10 | 35.90 | 1232.10 | 32779 | 71.00 | 32.60 | 0.88 | 98 | 52.50 | 130 | 0.82 | 0.20 | 66.80 | 43.40 | 42.60 |
| Sweden | 1.49 | 0.05 | 27.30 | 511.00 | 46251 | 81.70 | 0.20 | 0.91 | 97 | 60.30 | 53 | 0.89 | 1.20 | 86.50 | 18.30 | 19.80 |
| United Kingdom | 1.50 | 0.18 | 32.60 | 2618.90 | 37931 | 25.00 | 0.80 | 0.91 | 93 | 55.70 | 137 | 0.89 | 1.10 | 99.80 | 11.60 | 14.80 |
| **Average** | **1.86** | **0.10** | **31.28** | **876.12** | **39282.20** | **59.07** | **5.51** | **0.89** | **87.67** | **52.82** | **96.80** | **0.86** | **0.78** | **82.41** | **23.03** | **22.37** |
| **Global** | **4.21** | **0.28** | **34.42** | **958.17** | **22402.49** | **48.76** | **10.81** | **0.77** | **87.85** | **50.52** | **165.47** | **0.73** | **1.55** | **64.74** | **19.97** | **17.33** |

Any italicised values indicate additional notes on the source of the data. Further details can be found in Table S1.

**Table S4.** Heterogeneity statistics for country-level chronic hepatitis B virus prevalence in people who inject drugs (grouped by UNAIDS region)

| **Country of report** | **I^2^ Statistic** |
| --- | --- |
| **Australasia** | |
| Australia | NA |
| **East and Southeast Asia** | |
| China | 97.80% |
| Korea (Republic of) | NA |
| Myanmar | NA |
| Thailand | NA |
| Vietnam | 82.40% |
| **Eastern Europe** | |
| Azerbaijan | 68.30% |
| Belarus | 97.50% |
| Bosnia and Herzegovina | NA |
| Bulgaria | 91.50% |
| Estonia | NA |
| Hungary | 54.90% |
| Latvia | 53.70% |
| Lithuania | NA |
| Moldova (Republic of) | 92.40% |
| Romania | NA |
| Slovakia | 0.00% |
| Ukraine | NA |
| **Middle East and North Africa** | |
| Cyprus | 49.10% |
| Israel | NA |
| Lebanon | NA |
| Saudi Arabia | NA |
| Syrian Arab Republic | NA |
| Tunisia | NA |
| Turkey | 93.70% |
| **North America** | |
| United States of America | NA |
| **South Asia** | |
| Afghanistan | 33.30% |
| Bangladesh | NA |
| India | 91.00% |
| Iran (Islamic Republic of Iran) | 98.20% |
| Maldives | NA |
| Nepal | 87.80% |
| Pakistan | NA |
| **Sub-Saharan Africa** | |
| Côte d'Ivoire | NA |
| Kenya | NA |
| Madagascar | NA |
| Mauritius | NA |
| Nigeria | NA |
| Seychelles | NA |
| Tanzania (United Republic of) | NA |
| **Western Europe** | |
| Austria | 0.00% |
| Belgium | 0.00% |
| Croatia | NA |
| Denmark | NA |
| France | NA |
| Germany | 0.00% |
| Greece | 44.50% |
| Luxembourg | NA |
| Netherlands | 41.40% |
| Norway | 6.50% |
| Portugal | 91.00% |
| Serbia | NA |
| Spain | NA |
| Sweden | NA |
| United Kingdom | NA |

NA, not applicable.

**Figures S2a-h. Forest plots**

Random-effects meta-analysis of HbsAg prevalences stratified by UNAIDS region.

**Figure a.** Australasia (n=1)


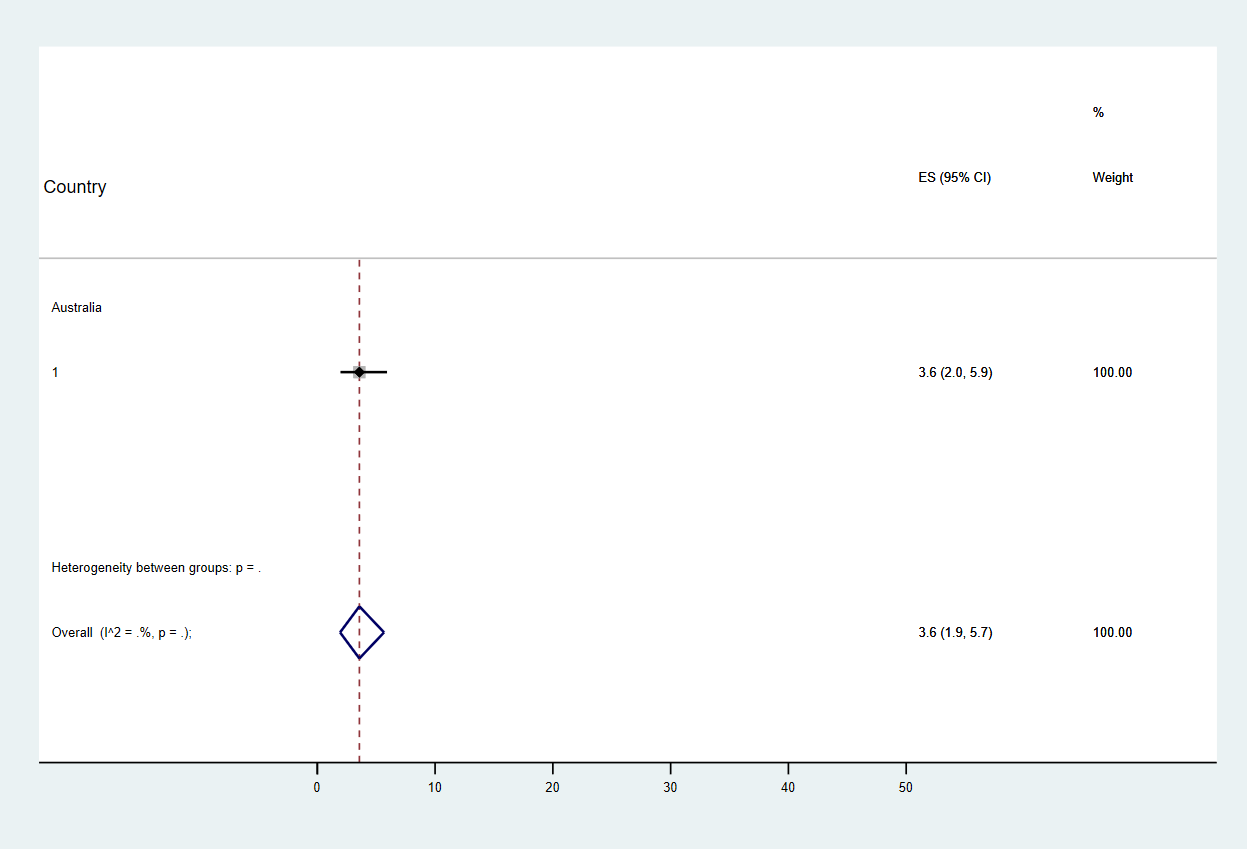


**Figure b.** East and Southeast Asia (n=5)


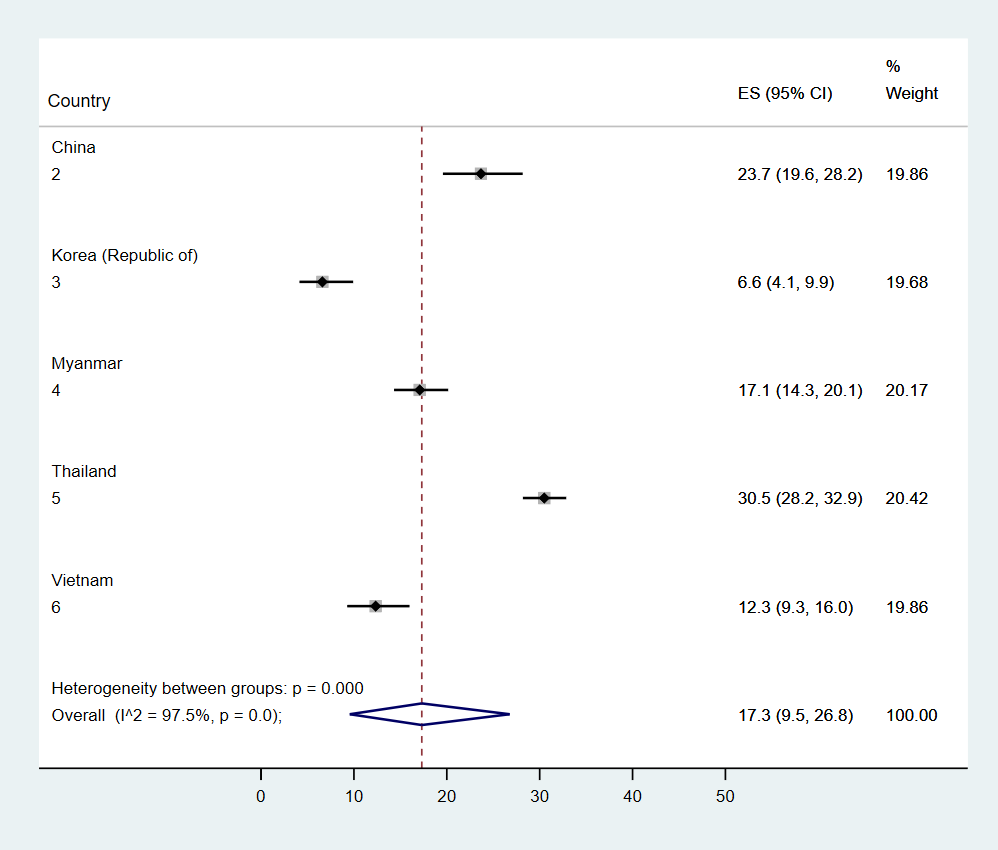


**Figure c.** Eastern Europe (n=12)


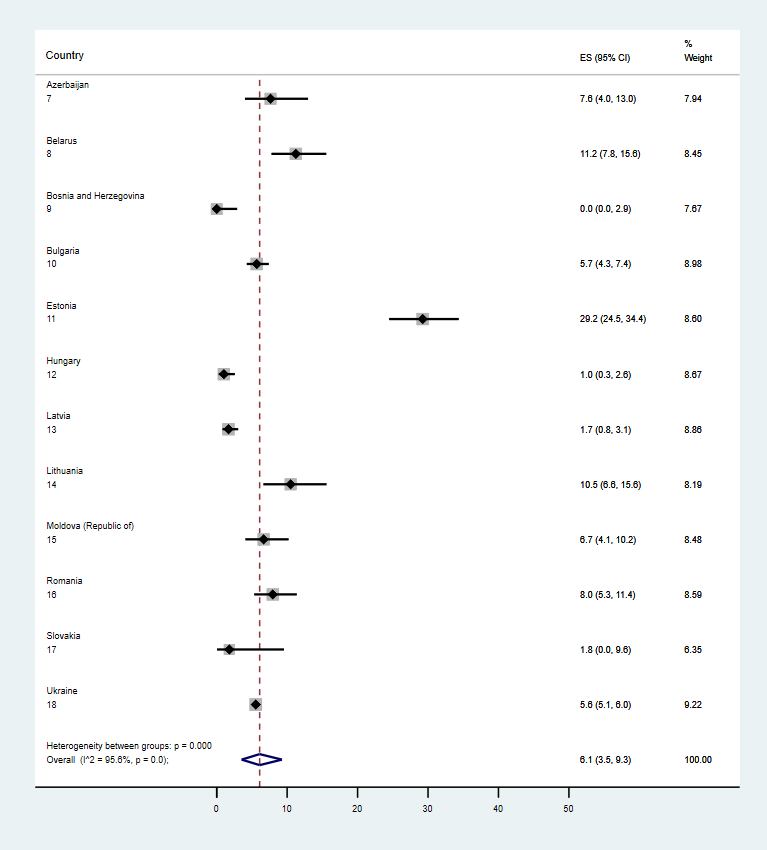


**Figure d.** Middle East and North Africa (n=7)


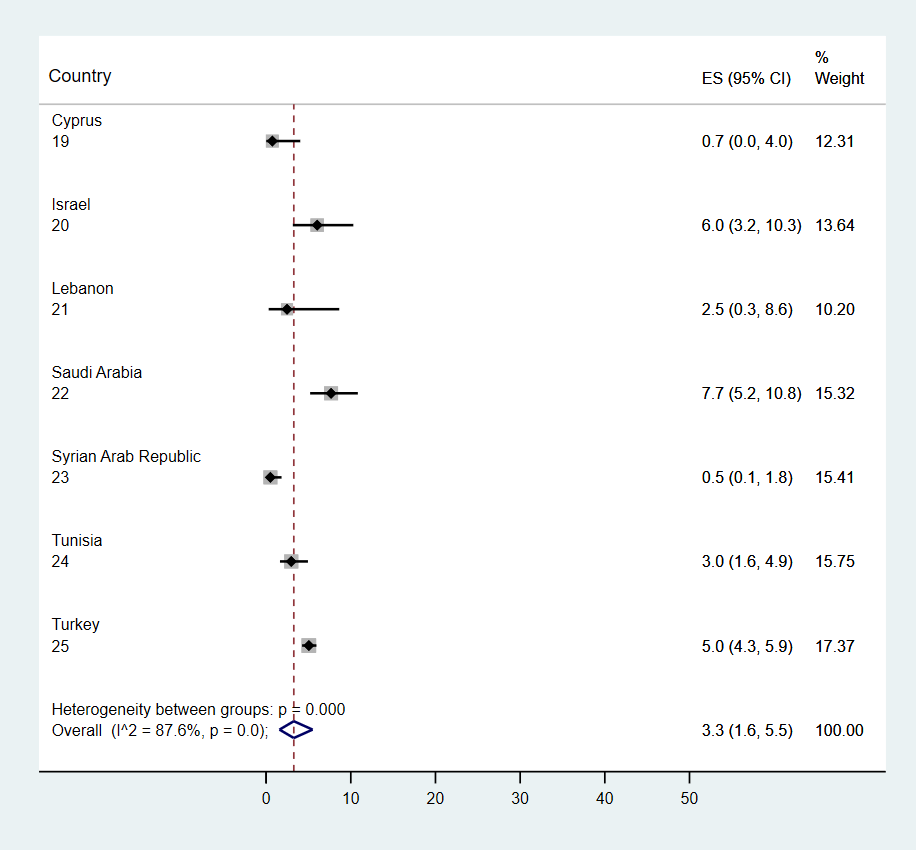


**Figure e.** North America (n=1)


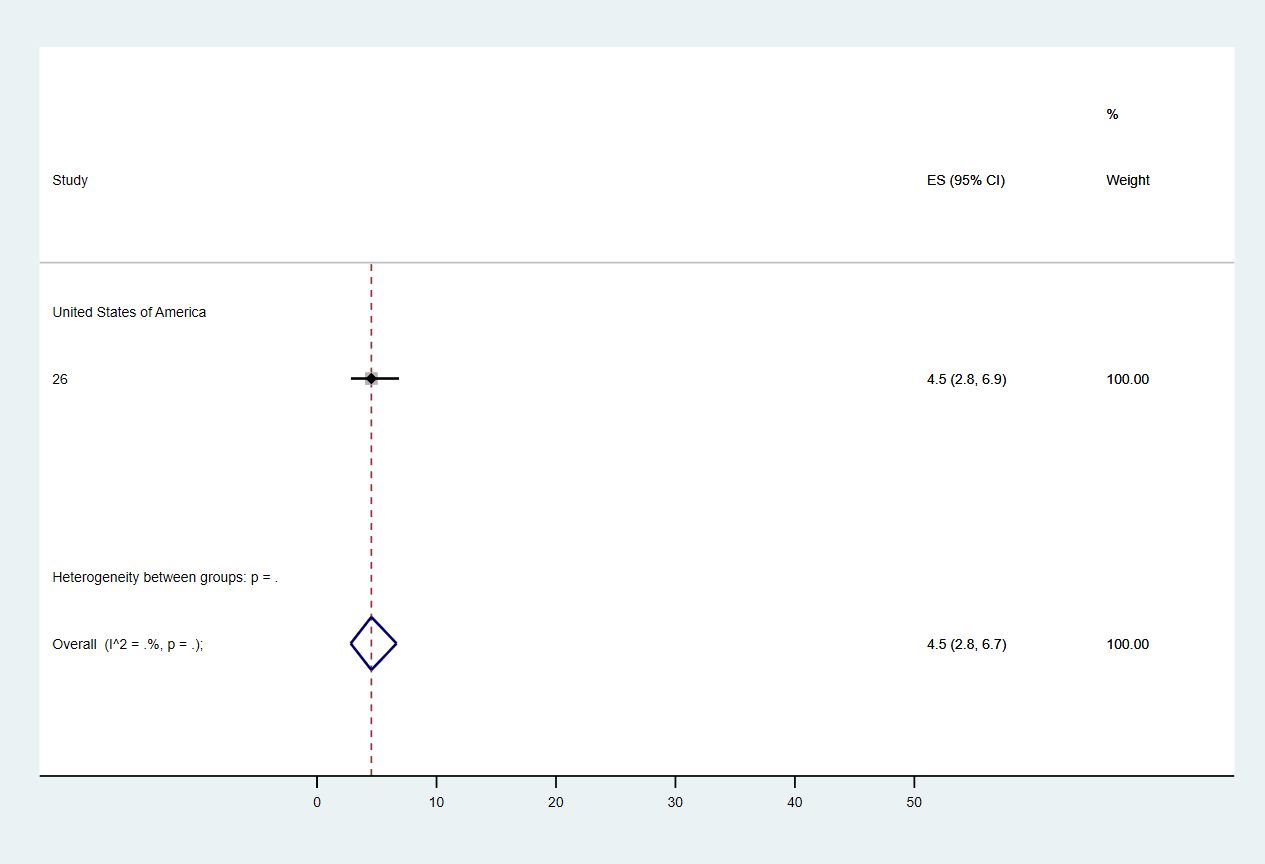


**Figure f.** South Asia (n=7)


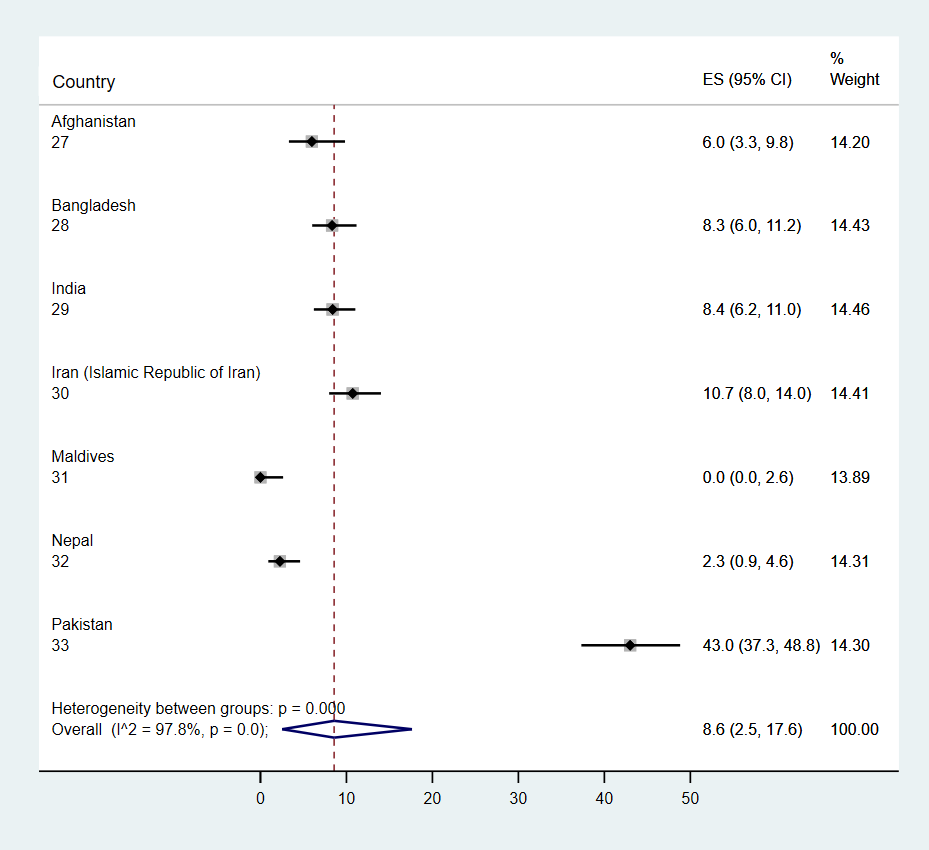


**Figure g.** Sub-Saharan Africa (n=7)


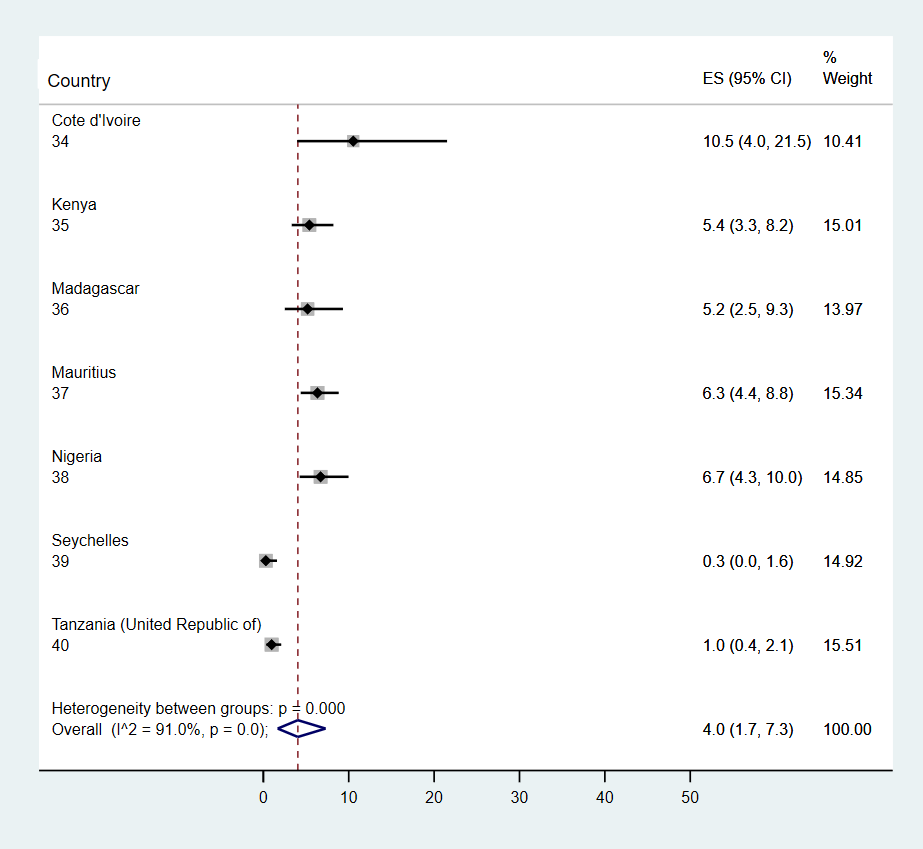


**Figure h.** Western Europe (n=15)


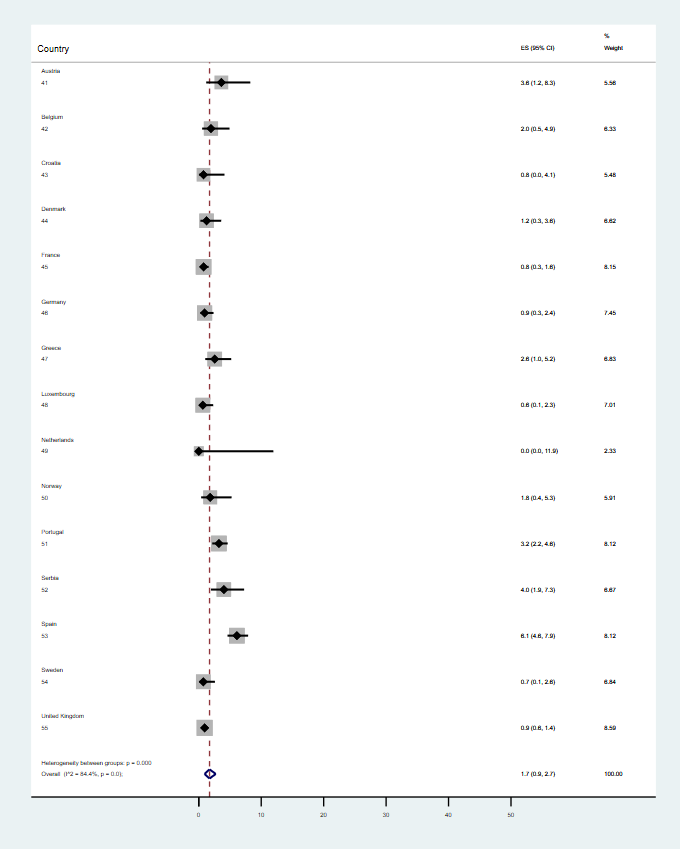


**Supplementary References**

1. Global Human Development Indicators: Technical Notes. United Nations Development Programme; 2020.
2. Global Human Development Indicators: Country Reports [Internet]. 2020. Available from: <http://hdr.undp.org/en/countries>.
3. The World Factbook: Gini Index coefficient – distribution of family income [Internet]. 2021. Available from: <https://www.cia.gov/the-world-factbook/field/gini-index-coefficient-distribution-of-family-income/country-comparison>.
4. Fernando J. Gross Domestic Product (GDP): Investopedia [updated 16/07/2021]. Available from: <https://www.investopedia.com/terms/g/gdp.asp>.
5. Syria GDP: Trading Economics; 2019. Available from: https://tradingeconomics.com/syria/gdp.
6. Cheung M. Gross National Income (GNI): Investopedia [updated 29/08/2021]. Available from: <https://www.investopedia.com/terms/g/gdp.asp>.
7. World Health Organisation (WHO) Regional Office for Europe. Hepatitis B 2021. Available from: <https://www.euro.who.int/en/health-topics/disease-prevention/vaccines-and-immunization/vaccine-preventable-diseases/hepatitis-b>.
8. Hayes A. Labour Force Participation: Investopedia; [updated 04/07/2021. Available from: <https://www.investopedia.com/terms/p/participationrate.asp#:~:text=What%20Is%20the%20Labor%20Force,%2C%20civilian%20working%2Dage%20population>.
9. Seychelles - Labor Force Participation Rate, Total (% Of Total Population Ages 15+) (national Estimate): Trading Economics; 2021. Available from: <https://tradingeconomics.com/seychelles/labor-force-participation-rate-total-percent-of-total-population-ages-15-national-estimate-wb-data.html>.
10. Socio-Demographic Index (SDI): Institute for Health Metrics and Evaluation (IHME); 2021 [updated 16/08/2021. Available from: <http://ghdx.healthdata.org/record/ihme-data/gbd-2019-socio-demographic-index-sdi-1950-2019>.
11. School enrollment, secondary, female (% gross) - Madagascar [Internet]. The World Bank. 2020. Available from: <https://data.worldbank.org/indicator/SE.SEC.ENRR.FE?locations=MG>.
12. Nigeria - School Enrollment, Secondary (% Gross): Trading Economics; 2021. Available from: <https://tradingeconomics.com/nigeria/school-enrollment-secondary-percent-gross-wb-data.html>.
13. Seychelles - Unemployment, Youth Female (% Of Female Labor Force Ages 15-24) (national Estimate): Trading Economics; 2021. Available from: <https://tradingeconomics.com/seychelles/unemployment-youth-female-percent-of-female-labor-force-ages-15-24-national-estimate-wb-data.html>.
14. Seychelles - Unemployment, Youth Male (% Of Male Labor Force Ages 15-24) (national Estimate): Trading Economics; 2021. Available from: <https://tradingeconomics.com/seychelles/unemployment-youth-male-percent-of-male-labor-force-ages-15-24-national-estimate-wb-data.html>.
15. World Development Indicators [Internet]. The World Bank. 2021. Available from: <https://datacatalog.worldbank.org/dataset/world-development-indicators>.
16. Global Burden of Disease Study (GBD) [Internet]. 2019. Available from: <http://ghdx.healthdata.org/gbd-2019>.
17. The Global Health Observatory [Internet]. 2021. Available from: <https://www.who.int/data/gho/data/indicators>.
